# Asymptomatic Periprosthetic Joint Infection of the Hip with High-Virulence Pathogens: Report of Two Cases

**DOI:** 10.1155/2022/2699779

**Published:** 2022-09-19

**Authors:** Ruben A. Mazzucchelli, Christoph Meier, Yvonne Achermann, Peter Wahl

**Affiliations:** ^1^Division of Orthopaedics and Traumatology, Cantonal Hospital Winterthur, Winterthur 8401, Switzerland; ^2^Division of Infectious Diseases and Hospital Epidemiology, University Hospital Zurich, Zurich 8091, Switzerland

## Abstract

Periprosthetic joint infection (PJI) may be a life-threatening condition, particularly when caused by pathogens with high virulence, capable of developing secondary bloodstream infection. We report two cases of chronic PJI of the hip, one with *Staphylococcus aureus* in a 27-year-old female with severe anorexia, the other one with *Staphylococcus lugdunensis* in a 74-year-old female suffering from morbid obesity. Both infections did not cause relevant symptoms over time despite the absence of suppressive antibiotic treatment. To our knowledge, there are no similar cases described in the literature. While it remains difficult to recommend postponing treatment in such cases, this option may be an alternative to suppressive antibiotic therapy.

## 1. Introduction

Periprosthetic joint infections (PJI) represent a great challenge in terms of diagnostics, treatment, and healthcare resources [1–3]. Cases are rising alongside the increasing number of patients undergoing arthroplasty in the first world [4]. Roughly, acute PJI is caused mainly by high-virulence pathogens like *S. aureus*, can be differentiated from subacute or chronic PJI, typically becoming manifest months or years after the index operation and involving low-virulence microorganisms such as coagulase-negative staphylococci, among others [5]. In both cases, patients experience pain in the affected joint. In the acute setting, the pain is caused by the inflammatory process, whereas a chronic infection ultimately leads to symptomatic implant loosening. Wound breakdown or sinus tracts may develop. The latter are poorly tolerated by the affected patients [6]. State-of-the-art treatment of PJI involves surgical revision combined with adequate, specific antibiotic treatment, as a spontaneous cure of an implant-associated infection is not to be expected [2]. We report two cases of PJI after total hip arthroplasty (THA) caused by highly virulent pathogens, initially presenting classic symptoms that rapidly faded despite the absence of adequate surgical and antibiotic treatment. Both patients subsequently remained asymptomatic for years without radiological evidence of implant failure. To the best of our knowledge, there are no similar reports available in the literature.

## 2. Case Presentation

### 2.1. Case 1

**Figure 1:**
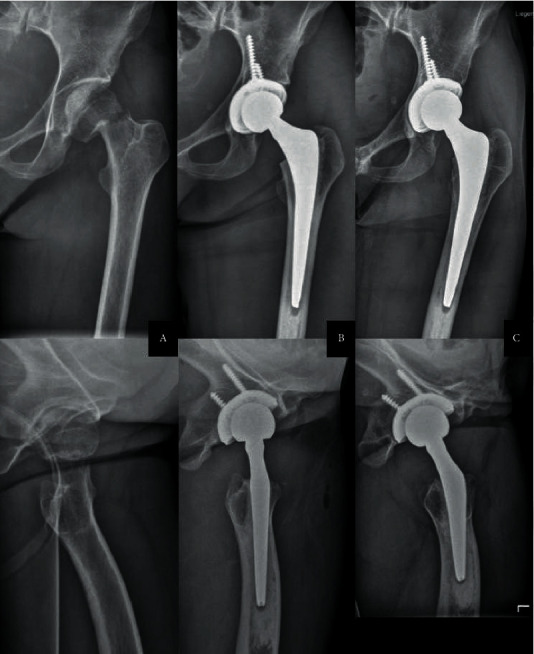
Anteroposterior and axial radiographs of the left hip from case one at the time of the dislocated stress fracture of the femoral neck (a) on the second postoperative day after hybrid THA (b) and at the one-year mark (c) Several months after untreated acute PJI with *S. aureus* the implants are well fixed, in particular, the cup is fully integrated. Note thinning out of the cancellous bone in the proximal femur as well as of the cortical bone on the femoral diaphysis, with an enlarged medullary cavity, indicating osteoporosis. The patient suffered from refractory anorexia nervosa.

A 27-year-old female patient with refractory anorexia nervosa (BMI of 16 kg/m^2^) developed avascular necrosis of the left femoral head after a neglected femoral neck stress fracture and ultimately required THA. Considering the severely reduced bone quality and the small anatomy of the hip, hybrid fixation was chosen, with the operation performed through a direct anterior approach (DAA). One month after surgery, she developed a fever, chills, and purulent wound discharge at the surgical site. Joint aspiration as well as blood cultures revealed growth of *S. aureus*. Unfortunately, leukocyte count was not available due to the low volume of aspiration. The patient, however, refused any surgical revision. Nevertheless, suppressive antibiotic treatment with amoxicillin was initially accepted and tolerated, but discontinued by the patient after only 3 weeks. 3 months later, the left hip was inconspicuous with no inflammatory signs, showing a normal range of motion, and the patient could weight-bear without any pain. The patients' anorexia was poorly controlled at the time, with polydipsia and consecutive severe hyponatraemia, impending liver failure, and recurrent ascites. The revision was therefore postponed due to these prohibitive general conditions and was later refused by the patient. Over the next months, no wound complications occurred, the fevers did not reappear and the patient continued to deny any symptoms on her left hip. Blood tests showed no signs of ongoing infection. In order not to compromise the fragile relationship with the patient, the interdisciplinary decision was to take no further action and observe the course of the events. After 6 months, the patient was still pain-free. Joint aspiration could be repeated and came back sterile with a low cell count (250 leukocytes/*µ*l). At one year, the patient was ambulating without discomfort and showing a normal gait. There were no clinical signs of infection. Conventional radiographs showed well-fixed components without signs of loosening (Figure 1). In particular, the cup seemed even to have reintegrated, the initial signs of loosening having disappeared. Now, at two years of follow-up, the patient still has no complaints.

### 2.2. Case 2

**Figure 2:**
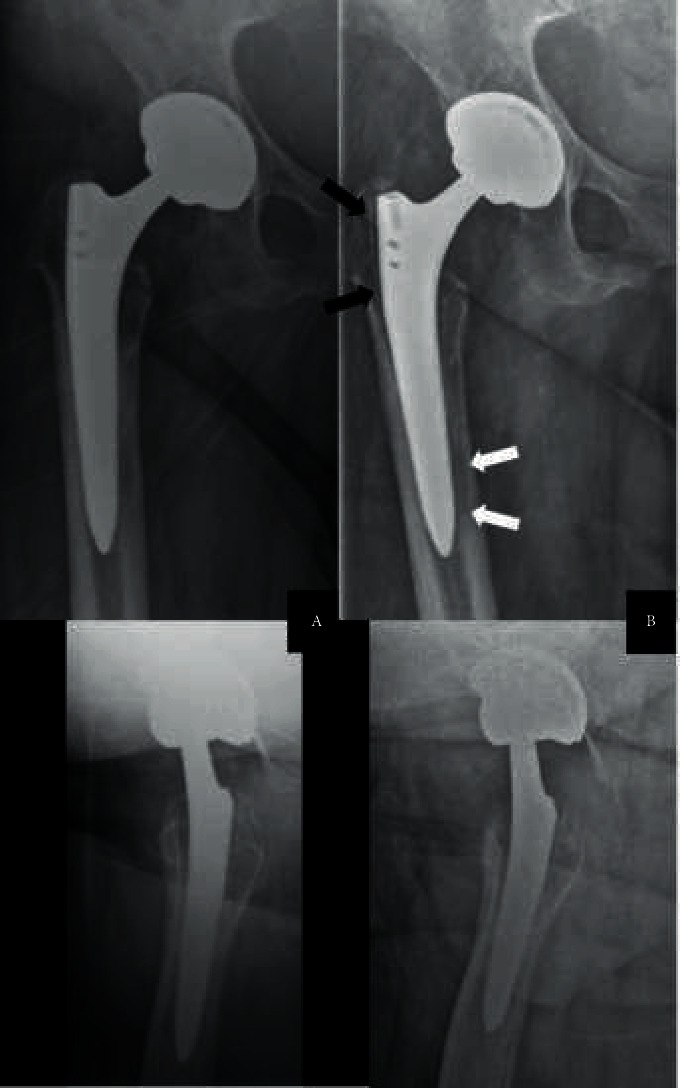
Anteroposterior and axial radiographs of the right hip of Case Two, 6 years after revision THA (a) Despite a newly diagnosed *S. lugdunensis* PJI, components show no signs of loosening. At the 10 years mark (b) after 4 years of documented, untreated PJI, the radiolucent line observable frequently in Gruen zone 1 with this type of stem (black arrows) remains stable. There is also some evidence of diaphyseal, endocortical osteolysis (white arrows), but no subsidence of the stem, and endosteal ossification at the tip of the stem remains stable. The cup is well fixed. In an asymptomatic patient, this implant can be considered sufficiently integrated. Note the poor contrast, particularly on the axial views in the lower row, due to morbid obesity (BMI of 48 kg/m^2^).

A 74-year-old, morbidly obese (BMI of 48 kg/m^2^) and diabetic female patient underwent uncemented THA over a DAA elsewhere for symptomatic osteoarthritis. She developed acute polymicrobial PJI within 4 weeks, with cultures positive for *S. epidermidis*, *C. acnes*, and *M. morganii*. Initial debridement, antibiotics, and implant retention (DAIR) treatment failed. Therefore, the patient underwent implant removal followed by several second-look procedures and vacuum-assisted wound therapy. No spacer had been used in the interval. The consecutive soft-tissue defect was covered with a pediculate infraumbilical rotation-transposition-advancement flap. Component reimplantation was performed 1 month later, respectively, four months after resection arthroplasty, with uncemented components over the same DAA. Further recovery apparently was uneventful. Six years later, the patient was referred to our department with persistent right-sided hip pain. Conventional radiographs did not show clear evidence of loosening, nor any periprosthetic fracture. Joint aspiration was performed showing pathological cell count (226′300 leukocytes/µl, 96% granulocytes) and grew *S. lugdunensis*. Revision surgery was scheduled but could not be performed due to an in-hospital fall causing the patient to sustain an open malleolar fracture necessitating multiple procedures and finally ankle fusion with a hindfoot nail followed by 12 weeks of oral amoxicillin and clavulanic acid. After 3 months, the patient had no symptoms on her right hip and was ambulating with a walker. Blood work was normal. Given her comorbidities and the lack of symptoms, an interdisciplinary decision was taken to indefinitely postpone revision, despite *S. lugdunensis* being a coagulase-negative *Staphylococcus* with high virulence. Now, 10 years after prosthetic component reimplantation and 4 years after the identification of the PJI, the patient still denies symptoms in her right hip. Conventional radiographs show a well-fixed cup. The uncemented stem shows a radiolucent line around its shoulder but has not subsided in 10 years and has no pedestal sign (Figure 2). In the absence of hip and/or thigh pain, we conclude that the implants are properly integrated and fixed and no further treatment is planned.

## 3. Discussion

The cases described above are neither representative for classic manifestation nor for state-of-the-art final management of PJI [1, 2]. Both patients suffer from severe comorbidities and can therefore be classified as Type C hosts [7, 8]. The overall complication rate for revision surgery for PJI should not be underestimated. In-hospital mortality for septic cases is twice as high as for aseptic revisions [9] and the 1-year mortality is significantly increased with an odds ratio of 3.6 according to a systematic review [10]. In a case-control study, the overall mortality of elderly patients undergoing two-stage revisions for PJI was as high as 40% over 5 years [11]. This should be taken into account, particularly when dealing with multimorbid patients requiring multiple surgical stages.

The first patient had developed early signs of an acute PJI with a classical high-virulence microorganism (*S. aureus*), which had subsequently chronified and eventually become completely asymptomatic. In this case, early revision (DAIR) according to guidelines [1, 2] could not be performed as planned due to patient refusal and extremely high perioperative risk due to impending liver failure. It appears that despite having all the risk factors for a compromised immune system, this infection remains silent and has not influenced the integration of the cup. Extreme denaturation and liver failure could be an explanation for the insufficient inflammatory response [12], and therefore, the low cell count in the joint aspiration. Furthermore, follow-up visits will be necessary to determine if this PJI remains asymptomatic in the future.

In the second case, we were facing a typical history of chronic infection after what appeared initially to be a successful two-stage revision for an early polymicrobial PJI after THA. The patient presented many risk factors for a PJI, such as obesity, diabetes, and chronic venous insufficiency. Female gender and heart disease are the main risk factors for PJI recurrence after two-stage revision THA [13]. In this case, the first PJI may be considered healed following a multistage revision with flap reconstruction of a soft-tissue defect, but a new PJI complicated the course of events. The fact that this recurrent PJI is caused by a new pathogen matches findings from a retrospective study showing that two thirds of recurrent PJI are caused by new pathogens [14]. *S. lugdunensis* belongs to the group of coagulase-negative staphylococci, known to be responsible for most of the low-grade, chronic PJI [15–17]. Nonetheless, *S. lugdunensis* can behave like a pathogen with high virulence, especially in the case of PJI, and should therefore not be underestimated [18]. The most appropriate treatment for this patient would consist of revision with component exchange, according to current guidelines [1, 2]. The unpredictable event of an in-hospital injury requiring multiple operations led us to postpone the initially planned revision. Surprisingly, the infection did not progress, and it still remains asymptomatic 4 years after the diagnosis was confirmed. Some radiological findings could warrant a discussion about whether the implant is well fixed or not. The radiolucent lines around the shoulder suggest some motion has occurred at some point. Thus said, the stem has not subsided in 10 years and the patient is ambulating without pain, which we interpret as the best clinical sign of sufficient osteointegration.

Both PJIs reported have not been treated surgically following standard guidelines because of adverse clinical circumstances. When surgery is not a viable option or patients have a low life expectancy, suppressive antibiotic therapy can be considered. A recent publication presents the results of 26 patients treated with suppressive antibiotic therapy for PJI of the hip or the knee, describing a success rate of 84% at an average follow-up of 3 years [19]. In a multicenter cohort study of 136 elderly patients, suppressive treatment for PJI was considered a reasonable alternative despite having up to 33% of adverse drug reactions [20]. Surgical salvage procedures such as persistent fistulas are only very rarely indicated due to their poor mid-term outcome and very low patient satisfaction and can be therefore considered a relic from the past [6]. Maybe simple therapeutic abstinence, as in these two cases, maybe as successful as long-term suppressive therapy without risking the potentially severe adverse events associated with antibiotic treatment, with an incidence of at least 15% [20, 21].

In conclusion, the fact that two high-virulence pathogens can colonize implants over time without causing symptoms or mechanical failure is a rarity so far, and both cases need further observation in the future. Therapeutic abstinence, however, may be considered as an alternative to long-term antibiotic suppression in selected cases, despite not being mentioned as an option in any guideline so far.

## Data Availability

All data are available within the article.
